# Electrophysiological Variations in Auditory Potentials in Chronic Tinnitus Individuals: Treatment Response and Tinnitus Laterality

**DOI:** 10.3390/jcm14030760

**Published:** 2025-01-24

**Authors:** Ourania Manta, Dimitris Kikidis, Winfried Schlee, Berthold Langguth, Birgit Mazurek, Jose A. Lopez-Escamez, Juan Martin-Lagos, Rilana Cima, Konstantinos Bromis, Eleftheria Vellidou, Zoi Zachou, Nikos Markatos, Evgenia Vassou, Ioannis Kouris, George K. Matsopoulos, Dimitrios D. Koutsouris

**Affiliations:** 1 Biomedical Engineering Laboratory, School of Electrical and Computer Engineering, National Technical University of Athens, 15780 Athens, Greece; rmanta@biomed.ntua.gr (O.M.); konbromis@biomed.ntua.gr (K.B.); ebel@biomed.ntua.gr (E.V.); ikouris@biomed.ntua.gr (I.K.); gmatsopoulos@biomed.ntua.gr (G.K.M.); dkoutsou@biomed.ntua.gr (D.D.K.); 2 Cyberalytics Limited, Nicosia 2369, Cyprus; 3 1st Department of Otorhinolaryngology, Head and Neck Surgery, National and Kapodistrian University of Athens, Hippocrateion General Hospital, 15772 Athens, Greece; zoizachou@gmail.com (Z.Z.); markatosn84@gmail.com (N.M.); evassou@uoa.gr (E.V.); 4 Department of Psychiatry and Psychotherapy, University of Regensburg, 93053 Regensburg, Germany; winfried.schlee@ost.ch (W.S.); berthold.langguth@medbo.de (B.L.); 5 Institute for Information and Process Management, Eastern Switzerland University of Applied Sciences, 9001 St. Gallen, Switzerland; 6 Tinnitus Center, Charité—Universitätsmedizin Berlin, Freie Universität Berlin and Humboldt-Universität zu Berlin, 10117 Berlin, Germany; birgit.mazurek@charite.de; 7 Division of Otolaryngology, Department of Surgery, Facultad de Medicina, Universidad de Granada, Parque Tecnológico de la Salud, Av. de la Investigación, 11, 18016 Granada, Spain; jose.lopezescamez@sydney.edu.au (J.A.L.-E.); juanmartinlagos@hotmail.com (J.M.-L.); 8 Meniere’s Disease Neuroscience Research Program, Faculty of Medicine & Health, School of Medical Sciences, The Kolling Institute, University of Sydney, Sydney, NSW 2050, Australia; 9 Sensorineural Pathology Programme, Centro de Investigación Biomédica en Red en Enfermedades Raras (CIBERER), 28029 Madrid, Spain; 10 Department of Otorhinolaryngology, Hospital Clínico Universitario San Cecilio, Instituto de Investigación Biosanitaria, ibs.Granada, 18071 Granada, Spain; 11 Faculty of Psychology and Educational Science, Health Phycology, KU Leuven University, B-3000 Leuven, Belgium; rilana.cima@kuleuven.be

**Keywords:** tinnitus, chronic subjective tinnitus, auditory evoked potential, treatment response, tinnitus laterality, auditory brainstem response, auditory middle latency response, electrophysiological differences, statistical analysis, underlying mechanisms

## Abstract

**Background**: This study investigates electrophysiological distinctions in auditory evoked potentials (AEPs) among individuals with chronic subjective tinnitus, with a specific focus on the impact of treatment response and tinnitus localisation. **Methods**: Early AEPs, known as Auditory Brainstem Responses (ABR), and middle AEPs, termed Auditory Middle Latency Responses (AMLR), were analysed in tinnitus patients across four clinical centers in an attempt to verify increased neuronal activity, in accordance with the current tinnitus models. Our statistical analyses primarily focused on discrepancies in time–domain core features of ABR and AMLR signals, including amplitudes and latencies, concerning both treatment response and tinnitus laterality. **Results**: Statistically significant differences were observed in ABR wave III and V latencies, ABR wave III peak amplitude, and AMLR wave Na and Nb amplitudes when comparing groups based on their response to treatment, accompanied by varying effect sizes. Conversely, when examining groups categorised by tinnitus laterality, no statistically significant differences emerged. **Conclusions**: These results provide valuable insights into the potential influence of treatment responses on AEPs. However, further research is imperative to attain a comprehensive understanding of the underlying mechanisms at play.

## 1. Introduction

Tinnitus, defined as the perception of a phantom sound and the emotional reaction to it, is a prevalent auditory symptom affecting a substantial portion of the global population [1,2]. Subjective tinnitus is the most common form, wherein the perceived sound is only heard by the affected individual [2,3]. The impact of tinnitus on patients can range from mild annoyance to severe distress, particularly when comorbidities are present [4]. While tinnitus is not often linked to any specific medical cause and lacks a definitive cure [5,6], it presents a complex and heterogeneous condition with varying frequencies, intensities, and durations [7]. Moreover, tinnitus can affect individuals of all ages, making it a diverse and challenging condition to study [7].

Epidemiological studies have revealed that tinnitus affects more than 10% of the general population, with approximately 1% of individuals considering it their most serious health concern [8,9,10]. Moreover, the prevalence of tinnitus is expected to increase significantly in the coming decades [8,9,10]. Despite significant scientific advancements in recent years, many tinnitus sufferers remain undertreated or receive inadequate management [1]. This situation leads to increased complaints, prolonged suffering, social withdrawal, excessive healthcare utilisation, and a complex network of referral pathways, imposing considerable psychological and financial burdens at both national and global levels [1]. Consequently, tinnitus continues to present a scientific and clinical enigma [11,12].

An Auditory Evoked Potential (AEP) is an electrical signal that the brain generates in response to a precisely timed auditory stimulus presentation [13]. This AEP signal is an average of responses from multiple stimulus repetitions. AEPs offer a non-invasive and objective testing method known for its simplicity, reproducibility, and cost-effectiveness. Audiologists utilise AEP responses to identify potential neural pathway obstructions in the brain. Furthermore, AEPs are valuable in diagnosing or ruling out hearing impairments, especially in infants, and have legal and medical relevance for excluding conditions like benign acoustic neuromas originating from the acoustic nerves [14].

Auditory Evoked Potentials (AEPs) are grouped into early (Auditory Brainstem Responses: ABRs), middle (Auditory Middle Latency Responses: AMLRs), and late (Auditory Late Latency Responses: ALLRs) responses based on their timing after a triggering event [15]. A typical ABR signal displays five peaks labelled with Roman numerals (I–V), each corresponding to distinct auditory structures [16]. AMLR is characterised by four waves of interest (Na, Pa, Nb, and Pb), reflecting thalamic and auditory cortex activity [17]. These AEPs can provide valuable insights into potential auditory pathway dysfunctions and may help understand the underlying mechanisms of tinnitus [18]. It is widely accepted that AEPs are not yet part of routine tinnitus-related clinical evaluations, and their correlation with tinnitus pathophysiology remains underexplored [18]. Nonetheless, the objective data provided by AEPs could enhance the understanding of tinnitus pathophysiology, leading to a more evidence-based approach to its management [18].

Within the realm of tinnitus research, it is imperative to account not solely for the discomfort or distress experienced by patients but also for potential variations in tinnitus experiences arising from factors such as sex, age, hearing loss, tinnitus duration, and tinnitus laterality. Our study focused on investigating the potential influence of treatment response, measured by improvements in Tinnitus Handicap Inventory (THI) scores before and after treatment, on the core metrics characterising ABR and AMLR signals (latency and amplitude). In the second stage, we considered how tinnitus laterality categories, including unilateral tinnitus–non-tinnitus ear, unilateral tinnitus–tinnitus ear, bilateral tinnitus ears, and head-localised tinnitus, impact these core ABR and AMLR signal metrics.

Based on existing research into auditory evoked potentials (AEPs) and their potential to reflect neural activity patterns, we hypothesise that individuals with chronic subjective tinnitus may exhibit distinct electrophysiological profiles. This would also be an indirect way to endorse the most currently adapted tinnitus models, which hypothesise additional neural gain in the auditory cortex and subcortical auditory structures as the most possible parameters inducing tinnitus occurrence. Specifically, we expect that variations in AEP latencies and amplitudes may serve as phenotypic markers, potentially differentiating those with greater treatment responsiveness and providing insights into how tinnitus laterality influences auditory pathway function.

## 2. Materials and Methods

### 2.1. Data Origin, Recruitment Process, and Patient Characteristics

Data for this study were collected from the tinnitus database of the European project “Unification of Treatments and Interventions for Tinnitus Patients” (UNITI) [19]. The study was funded by the EU, number: 848261, ClinicalTrials.gov number: NCT04663828. Ethics Committee Approval 20943/2020. UNITI aimed to develop a predictive computational model for optimal treatment selection based on specific patient parameters [19]. The data used in this study were obtained from a randomised clinical trial (RCT) conducted under the UNITI project (ClinicalTrials.gov Identifier: NCT04663828) [20]. Patients’ information was gathered from four European clinical centres: Hippocrateion General Hospital of Athens, Greece; Klinikum der Universitaet Regensburg, Germany; Charité—Universitaetsmedizin Berlin, Germany; and University of Granada Hospitals, Granada, Spain.

Patients were recruited based on predefined criteria established by the UNITI consortium (Table S1). Strict adherence to ethical guidelines ensured a fair and non-discriminatory recruitment process with no potential for harm or threat to participants’ physical or mental well-being. All patients signed informed consent. Monetary incentives were not offered to participants.

### 2.2. Electrophysiological Measurements

In this study, a total of 484 ABR waveforms and 434 AMLR waveforms were utilised. These waveforms were recorded and extracted using the Interacoustics Eclipse system (module EP25) [21], which was employed consistently across all clinical centers to ensure uniformity in data acquisition. This system provides the option to export the unprocessed measurements of the recorded AEPs into files formatted as .xml (Extensible Markup Language). The ABR waveform recordings were conducted utilising an acoustic click as the eliciting stimulus, administered at a repetition rate of 22 stimuli per second. This auditory stimulus was presented at distinct sound intensity levels of 80 dB nHL and 90 dB nHL. The ensuing physiological responses were subjected to meticulous signal conditioning, involving a high-pass filter set at 33 Hz, 6 dB/octave, and a low-pass filter set at 1500 Hz. The entire process was executed at a sampling rate of 30 kHz. Conversely, the AMLR waveform recordings entailed the application of a 2 kHz tone-burst stimulus, encompassing a duration equivalent to 28 cycles of sine waves. The presentation rate of this auditory stimulus was set at 6.1 bursts per second while maintaining a constant sound intensity level of 70 dB nHL. The subsequent recorded physiological signals were subjected to analogous signal conditioning procedures with a high-pass filter set at 10 Hz, 12 dB/octave, and a low-pass filter set at 1500 Hz. The sample rate for this particular recording paradigm was established at 3 kHz. A comprehensive exposition of the intricate stimulus parameters and the precise acquisition parameters pertinent to each respective auditory test can be meticulously referenced in Table S2.

The R programming language and relevant packages were utilised to process and visualise the exported.xml files containing the raw data. Waveforms were then annotated using two automated tools for AEP wave detection [22]. Annotated waveforms were manually checked for validity.

### 2.3. Descriptive and Statistical Analyses in the Time Domain and Waveform Categorisation

In this study, statistical analyses were performed to identify electrophysiological differences among tinnitus sufferers based on treatment response and tinnitus laterality. The metrics analysed included the amplitudes and latencies of the AEP waves of interest. A *p*-value of less than 0.05 was considered statistically significant. Descriptive analyses included mean scores with standard deviations (SDs) and medians for continuous variables. Tests used to detect statistically significant differences included the Mann–Whitney U-Test (non-parametric), the *t*-Test, Welch’s *t*-Test, and the Kruskal–Wallis Test (non-parametric). The selection of the statistical test for each comparison was determined based on the outcomes of quantile–quantile plots (Q–Q plots) and Shapiro–Wilk tests, utilised to assess the normal distribution, as well as the results of Levene’s tests, employed to examine variances. Effect sizes were calculated for cases with statistically significant differences, using different measures depending on the test employed. Specifically, effect sizes were calculated using r (r = 0.1 indicates a small effect; r = 0.3 indicates a medium effect; r = 0.5 indicates a large effect) for the Mann–Whitney U-Test and Cohen’s d (d = 0.2 indicates a small effect; d = 0.5 indicates a moderate effect; d = 0.8 indicates a large effect) for the *t*-Test and Welch’s *t*-Test. For the Kruskal–Wallis tests, post hoc pairwise comparisons were conducted using the Dunn–Bonferroni test to correct for multiple comparisons.

The categorisation of waveforms within two compared groups, namely “significant improvement” and “no improvement”, contingent upon the treatment response, was executed through a two-step process. Initially, all waveforms originating from ears in which tinnitus matching was not detected were systematically excluded from consideration. Subsequently, from the remaining waveforms, those affiliated with individuals who corresponded in the upper quartile of the Tinnitus Handicap Inventory (THI) difference (THI difference = THI score before treatment minus THI score after treatment), signifying substantial amelioration in the THI score, formed the “significant improvement group”. Conversely, waveforms were associated with individuals who corresponded in the lowest quartile of the THI difference, indicating a lack of change or even a deterioration in the THI score and constituting the “no improvement group”. This procedure was defined a priori to ensure consistency and methodological robustness in the grouping criteria.

In the subsequent analysis, wherein the categorisation of waveforms was predicated upon the tinnitus laterality of patients, we incorporated all available waveforms. The comparative cohorts encompassed the waveforms associated with individuals afflicted by bilateral tinnitus, those emanating from ears afflicted by unilateral tinnitus (specifically, the tinnitus-affected ears), those from ears unaffected by tinnitus (specifically, the non-tinnitus ears of the unilateral tinnitus patients), and those originating from cases of head-localised tinnitus.

In all analyses conducted, it was assumed that the variable of patients’ age did not exert influence on the outcomes. This assumption was substantiated by the homogeneity observed in the descriptive statistics of the compared groups regarding this characteristic, as presented in Tables S3–S6. Furthermore, an essential facet of our investigation entailed a rigorous assessment of the potential influence of hearing loss across the comparative groups. This scrutiny was indispensable in ensuring the fundamental homogeneity of the groups under examination. Our meticulous examination sought to identify any statistically significant differences in hearing loss metrics among the distinct cohorts. The detailed results of these comparative analyses are comprehensively documented in the Supplementary Materials, accessible in Tables S7–S12. In line with this rationale, to ascertain the comparability of the groups, we deemed it pertinent to elucidate the gender distribution along with the presence or absence of hearing loss within each cohort. Consequently, Figures S1–S4, presented in the Supplementary Materials, depict Sankey diagrams elucidating the distribution of male and female individuals, as well as their hearing status, across the analysed groups.

## 3. Results

In this section, we present a comprehensive overview of the results obtained from the extensive descriptive and statistical analyses conducted within the framework of the study. The figures showcased herein display the violin plots of the waveform metrics, encompassing both latencies and amplitudes, highlighting the metrics where statistically significant differences were observed. These visual representations serve as valuable tools for elucidating the intricate electrophysiological disparities observed among the study participants, particularly concerning treatment response and tinnitus laterality. The graphical depictions encapsulate the key findings derived from the comparisons, shedding light on the distinctive characteristics of the AEPs within various subgroups. Each violin plot affords insights into the temporal and magnitude variations of the waveforms’ core metrics, contributing to a comprehensive understanding of the electrophysiological disparities among chronic subjective tinnitus sufferers.

### 3.1. Correlation Between Treatment Response and the Latency and Amplitude Components of ABR and AMLR Waveforms

As already mentioned, to investigate the influence of treatment response on the latency and amplitude attributes of AEP waveforms in individuals with tinnitus, we conducted both descriptive and statistical analyses. The data in Table 1 provide a combined presentation of the descriptive and statistical information concerning the latencies and amplitudes of peaks I, III, and V of the compared groups. Similarly, Table 2 offers descriptive statistical data pertaining to the latencies and amplitudes of peaks Pa and Pb, as well as the latencies and amplitudes of troughs Na and Nb of the compared groups, along with the statistical differences identified for each of the aforementioned AMLR components.

**Figure 1:**
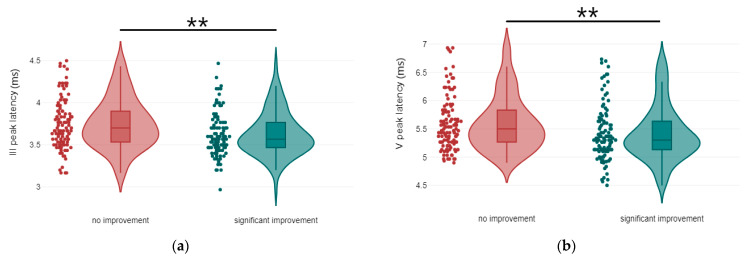
Violin plots of ABR latency components by treatment response: (**a**) III Peak latency, with significance indicated by asterisks (** *p*-value < 0.01); (**b**) V Peak latency, with significance indicated by asterisks (** *p*-value < 0.01).

**Figure 2:**
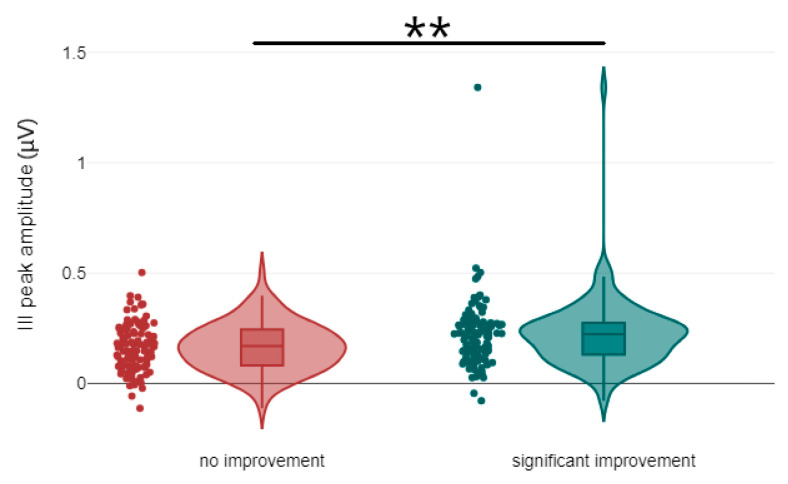
Violin plots of ABR amplitude components by treatment response. III Peak amplitude is shown, with significance indicated by asterisks (** *p*-value < 0.01).

**Figure 3:**
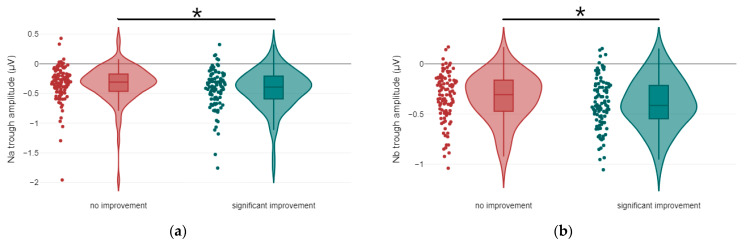
Violin plots of AMLR amplitude components by treatment response: (**a**) Na trough amplitude, with significance indicated by asterisks (* *p*-value < 0.05); (**b**) Nb trough amplitude, with significance indicated by asterisks (* *p*-value < 0.05).

Regarding ABR waveforms, significant differences were observed in peak ΙΙΙ and peak V latencies, as well as in peak IΙΙ amplitude (Figure 1 and Figure 2). For AMLR waveforms, Na trough and Nb trough amplitudes showed statistically significant differences (Figure 3).

### 3.2. Correlation Between Tinnitus Laterality and the Latency and Amplitude Components of ABR and AMLR Waveforms

To investigate the influence of tinnitus laterality on the latency and amplitude components of AEP waveforms, similar to the examination of treatment response, we conducted both descriptive and statistical analyses. In contrast with the treatment response groups, our investigation of tinnitus laterality groups’ homogeneity introduced a layer of complexity, as exemplified in ABR Tables S9 and S10 and AMLR Tables S11 and S12. The data in Table S13 provide a presentation of the descriptive statistical information concerning the latencies and amplitudes of peaks I, III, and V of the compared groups. Table S14 supplies details on the statistical differences that have been identified for each of the aforementioned ABR components. In a manner akin to the previous investigation, Table S15 offers descriptive statistical data pertaining to the latencies and amplitudes of peaks Pa and Pb, as well as the latencies and amplitudes of troughs Na and Nb of the compared groups. Correspondingly, Table S16 imparts information regarding the statistical differences detected for each of the aforementioned AMLR components.

**Figure 4:**
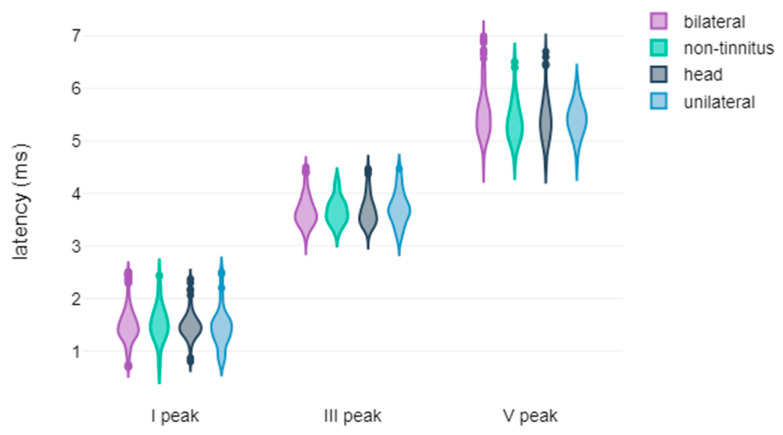
Violin plots of ABR waveform latencies based on tinnitus laterality.

**Figure 5:**
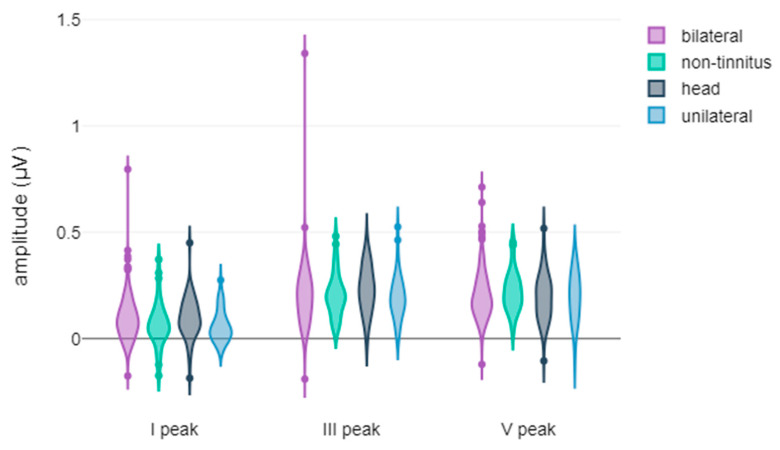
Violin plots of ABR waveform amplitudes based on tinnitus laterality.

**Figure 6:**
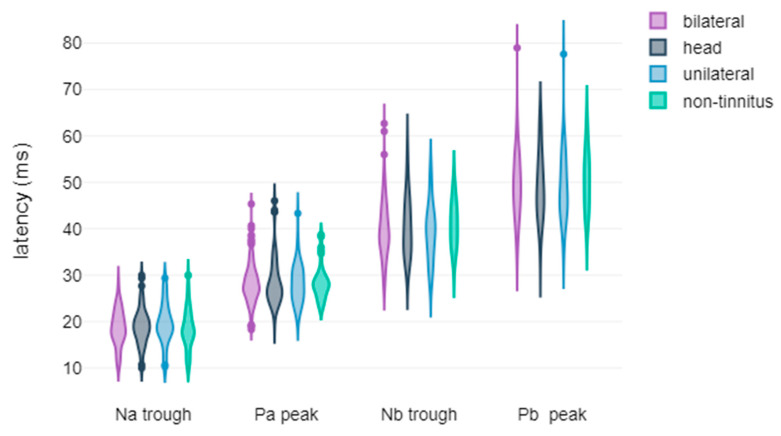
Violin plots of AMLR waveform latencies based on tinnitus laterality.

**Figure 7:**
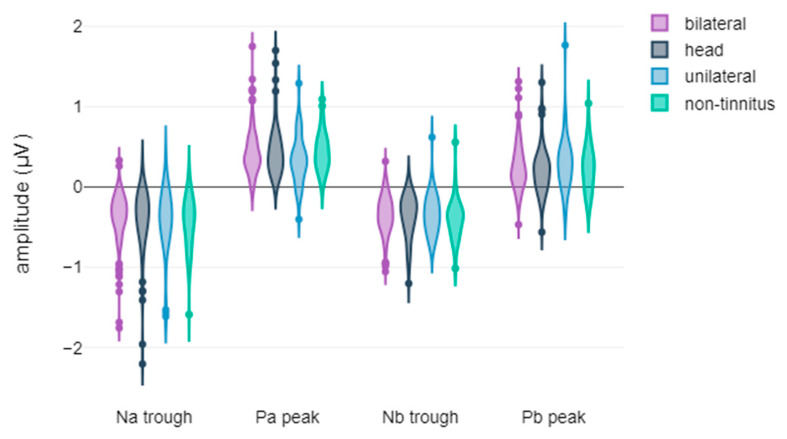
Violin plots of AMLR waveform amplitudes based on tinnitus laterality.

Figure 4, Figure 5, Figure 6 and Figure 7 illustrate violin plots representing the latencies and amplitudes of ABR and AMLR waveforms categorised by tinnitus localisation. Nevertheless, it is noteworthy that for both types of waveforms, our analysis did not reveal any statistically significant differences among the compared groups in any of the measured metrics.

## 4. Discussion

The present study delved into an in-depth investigation of the electrophysiological differences among chronic subjective tinnitus sufferers, with a particular focus on the influence of treatment response and tinnitus laterality. The comprehensive statistical analyses revealed noteworthy findings that shed light on the distinct patterns exhibited by auditory evoked potentials (AEPs), contrasting super responders (upper quartile THI score decrease) with non-responders (lower quartile THI score decrease), based on treatment response. In contrast, the analyses using tinnitus laterality as the categorisation criterion yielded results indicating the absence of statistical differences. These findings suggest a potential lack of influence of tinnitus laterality on the amplitudes and latencies of AEP signals.

Although a universally accepted and applied tinnitus treatment is still pending [1], one of the few points of global agreement in the tinnitus field is that a variant subgroup exists in different treatments with clinically meaningful responses. According to European guidelines, CBT, hearing aids, structured counselling and sound therapy have all been evaluated as beneficial and are recommended. Prognostic factors for treatment response based on patients’ profile, history and clinical characteristics would be really beneficial since it would optimise the use of resources. Hence, there is an absence of evidence in the literature about reliable prognostic factors, especially correlated with specific treatments. This study adhered to the European guidelines for tinnitus treatment, ensuring uniformity in patient care practices.

In the past, numerous anatomical sites were proposed as potential sources of tinnitus, including the auditory cortex, Heschl’s gyrus, inferior colliculus, cochlear nucleus, thalamus and medial geniculate body, limbic system, prefrontal cortex, cerebellum and superior frontal, as identified in a recent systematic review of fMRI studies in tinnitus [23]. Therefore, electrophysiology could be a robust, non-invasive way to identify neuronal activity in these areas and correlate these findings with tinnitus occurrence, properties, prognosis and course.

In a recent systematic review [24], only three studies correlating AMLR and tinnitus were identified. None of them used AMLR as potential treatment response factors, whereas only two of them focused on all waveforms (Na, Pa, Nb and Pb). All three studies evaluated the differences between tinnitus patients with normal hearing and controls. Only in one out of three studies a statistically significant difference was identified regarding the amplitudes of Na and Pa waves, which were found larger in tinnitus patients rather than in controls [25].

Our analysis of tinnitus laterality in relation to AEPs did not yield intriguing outcomes, as statistically significant differences were not detected in any cases. However, future investigations with larger sample sizes and more refined categorisations may provide deeper insights into the interplay between tinnitus laterality and electrophysiological responses.

ABR waves III and V latencies, ABR wave III amplitudes, as well as AMLR waves Na and Nb amplitudes, exhibited meaningful statistical differences between treatment response subgroups. The discernible effect sizes indicate that the identified variations are statistically significant and may hold clinical relevance. However, these findings should be interpreted with caution, as they represent a preliminary step and require confirmation in independent samples. Nonetheless, they highlight the potential utility of AEPs as objective measures for exploring the severity and characteristics of tinnitus, which could inform tailored treatment strategies for individual patients in the future.

In light of the supplementary analyses conducted to assess the homogeneity of our compared groups with regard to hearing status, we can further refine our interpretation of the primary findings. For the dimension of treatment response, where no statistically significant differences were identified in hearing status (Tables S7 and S8), the robust homogeneity across the groups not only bolsters the foundation of our primary analyses but also adds weight to the trustworthiness and validity of the insights we garnered. This reaffirms the potential utility of AEPs as objective markers of treatment response in individuals grappling with chronic subjective tinnitus.

However, our exploration of tinnitus laterality, where statistically significant differences in hearing status emerged (Tables S9–S12), introduces a nuanced dimension. The variations in hearing loss observed among the tinnitus laterality groups, particularly in the contrast between head-localised vs. unilateral-localised and unilateral vs. non-tinnitus groups (Tables S10 and S12) are totally predictable since hearing loss and tinnitus are strongly correlated approximately 90% of tinnitus patients also suffer from hearing loss); however, this factor is significant regarding data interpretation. These findings emphasise the intricate interplay between hearing status, tinnitus laterality, and AEPs.

It is worth noting that in our study, the compared groups exhibit analogous gender distributions, both in terms of the presence or absence of hearing loss. Consequently, we infer that these groups are reasonably homogenous with regard to these demographic factors. Nevertheless, in forthcoming studies featuring a larger cohort of normal-hearing participants, we recommend conducting separate analyses for those with and without hearing loss. This approach would effectively eliminate any potential influence of hearing loss as a confounding factor and enhance the precision of result interpretation.

We recognise that different treatments may have distinct mechanisms of action, potentially leading to specific alterations in AEPs associated with treatment response. A small number of participants per group did not allow powerful analysis.

It is essential to acknowledge certain limitations in our study. Specific inclusion criteria may have influenced the homogeneity of the study cohort [26]. Not all patients underwent the same treatment since they were randomised in groups receiving CBT, hearing aids, structured counselling, sound therapy and their possible combinations. Moreover, the cross-sectional nature of the investigation warrants caution in attributing causality to the observed associations [27,28]. To address these limitations and corroborate our findings, future longitudinal studies with diverse and larger participant cohorts are warranted. Another limitation was that potential confounders like high-frequency audiometry symptom duration and age were not taken into consideration.

Auditory evoked potentials (AEPs) hold promise as potential biomarkers for treatment response [24,29], particularly when combined with other datasets using advanced analytical methods such as artificial intelligence (AI) and decision support systems (DSS) [12,30]. These approaches could help identify complex patterns and improve the predictive accuracy of treatment outcomes in tinnitus patients. Furthermore, incorporating fMRI sessions into future research may provide a more detailed understanding of the insufficiently elucidated phenomenon of tinnitus, offering additional insights into the neural mechanisms involved. Future studies should explore the integration of AEP data with these methods to enhance their utility as biomarkers and deepen our understanding of tinnitus.

## 5. Conclusions

In conclusion, our study provides valuable insights into the electrophysiological differences among chronic subjective tinnitus sufferers with regard to treatment response and tinnitus laterality. Our findings revealed statistically significant differences in the amplitudes and latencies of specific AEP waveforms, including ABR wave III and V latencies, ABR wave III peak amplitude, and AMLR wave Na and Nb amplitudes, in relation to treatment response. While comparisons were made regarding tinnitus laterality, no statistically significant differences were identified in these analyses. By elucidating the elesiological correlates of tinnitus, our findings hold the potential to enhance our understanding of this complex condition and its underlying mechanisms. We advocate for future studies to build upon our findings, encompassing longitudinal designs and larger, diverse cohorts, to further advance the field and facilitate evidence-based clinical decision-making [31].

## Figures and Tables

**Table 1:** Descriptive and statistical analysis of the effect of treatment response on the latency and amplitude components of ABR waveforms. SD: Standard Deviation; # = Number of valid values.

	Treatment Response	Mean	SD	#	Mann–Whitney U-Test	Effect Size (r)
I peak latency	no improvement	1.53	0.42	109	U = 5384, *p* = 0.98, r = 0	too small
	significant improvement	1.54	0.38	99		
**III peak** **latency**	**no improvement**	**3.75**	**0.3**	**109**	U = 4113, ***p* = 0.001,** **r = 0.22**	medium
	**significant improvement**	**3.63**	**0.26**	**102**		
**V peak** **latency**	**no improvement**	**5.6**	**0.48**	**113**	U = 4746, ***p* = 0.004,** **r = 0.19**	small to medium
	**significant improvement**	**5.42**	**0.49**	**108**		
I peak amplitude	no improvement	0.07	0.09	109	U = 4547.5, *p* = 0.051, r = 0.14	small to medium
	significant improvement	0.11	0.12	99		
**III peak** **amplitude**	**no improvement**	**0.16**	**0.11**	**109**	U = 4183, ***p* = 0.002,** **r = 0.21**	medium
	**significant improvement**	**0.22**	**0.16**	**102**		
V peak amplitude	no improvement	0.18	0.11	113	U = 5371, *p* = 0.125, r = 0.1	small
	significant improvement	0.2	0.14	108		

**Table 2:** Descriptive and Statistical Analysis of the Effect of Treatment Response on the Latency and Amplitude Components of AMLR Waveforms. *SD: Standard Deviation; # = Number of valid values*.

AEP Component	Treatment Response	Mean	SD	#	Mann–Whitney U-Test or *t*-Test for Independent Samples	Effect Size (r or d)
Na trough latency	no improvement	18.85	4.4	102	U = 4719.5, *p* = 0.852, r = 0.01	too small
	significant improvement	19.17	3.73	94		
Pa peak latency	no improvement	28.65	4.89	103	U = 4701, *p* = 0.551, r = 0.04	too small
	significant improvement	28.15	3.93	96		
Nb trough latency	no improvement	40.3	6.78	103	U = 4824, *p* = 0.769, r = 0.02	too small
	significant improvement	40.13	6.28	96		
Pb peak latency	no improvement	49.51	8.27	103	t(197) = 0.26, *p* = 0.797, 95% CI [−1.87, 2.42] Cohen’s d = 0.04 variance equality	too small
	significant improvement	49.23	6.88	96		
**Na trough** **amplitude**	**no improvement**	**−0.33**	**0.31**	**102**	U = 3871.5, ***p* = 0.02,** **r = 0.17**	small to medium
	**significant improvement**	**−0.43**	**0.34**	**94**		
Pa peak amplitude	no improvement	0.39	0.26	103	U = 4340, *p* = 0.138, r = 0.11	small to medium
	significant improvement	0.44	0.29	96		
**Nb trough** **amplitude**	**no improvement**	**−0.33**	**0.24**	**103**	U = 4108.5, ***p* = 0.04,** **r = 0.15**	small to medium
	**significant improvement**	**−0.39**	**0.25**	**96**		
Pb peak amplitude	no improvement	0.21	0.24	103	U = 4309, *p* = 0.118, r = 0.11	small to medium
	significant improvement	0.27	0.35	96		

## Data Availability

The data presented in this study are available upon reasonable request from the corresponding author.

## Supplementary Materials

The following supporting information can be downloaded at: https://www.mdpi.com/article/10.3390/jcm14030760/s1, Table S1: Inclusion and exclusion criteria of the UNITI’s project RCT; Table S2: Stimulus and acquisition parameters for ABR and AMLR recordings; Table S3: Subjects’ age in the two treatment response groups of the ABR waveforms; Table S4: Subjects’ age in the two treatment response groups of the AMLR waveforms; Table S5: Subjects’ age in the four tinnitus laterality groups of the ABR waveforms; Table S6: Subjects’ age in the four tinnitus laterality groups of the AMLR waveforms; Table S7: Statistical hearing status analysis in two ABR waveform treatment response groups; Table S8: Statistical hearing status analysis in two AMLR waveform treatment response groups; Table S9: Statistical hearing status analysis in four ABR waveform tinnitus laterality groups; Table S10: Statistical hearing status analysis in four ABR waveform tinnitus laterality groups (Dunn–Bonferroni tests); Table S11: Statistical hearing status analysis in four AMLR waveform tinnitus laterality groups; Table S12: Statistical hearing status analysis in four AMLR waveform tinnitus laterality groups (Dunn–Bonferroni tests); Table S13: Descriptive statistics regarding the effect of tinnitus laterality on the latency and amplitude components of the ABR waveforms; Table S14: Statistical differences regarding the effect of tinnitus laterality on the latency and amplitude components of the ABR waveforms; Table S15: Descriptive statistics regarding the effect of treatment response on the latency and amplitude components of the AMLR waveforms; Table S16. Statistical differences regarding the effect of treatment response on the latency and amplitude components of the AMLR waveforms, Figure S1: Gender and hearing loss distribution in ABR waveform groups—treatment response; Figure S2: Gender and hearing loss distribution in AMLR waveform groups—treatment response; Figure S3: Gender and hearing loss distribution in ABR waveform groups—tinnitus laterality; Figure S4: Gender and hearing loss distribution in AMLR waveform groups—tinnitus laterality.
