# Supplementary material for: Electrophysiological Variations in Auditory Potentials in Chronic Tinnitus Individuals: Treatment Response and Tinnitus Laterality
Source: J Clin Med. 2025 Jan 24;14(3):760. doi: 10.3390/jcm14030760 (PMC11818601; doi:10.3390/jcm14030760)
Supplement: Supplementary file 1 [file jcm-14-00760-s001.zip › jcm-3374260-supplementary.pdf]

Table S1. Inclusion and exclusion criteria of the UNITI's project RCT.

**Inclusion Criteria**
Tinnitus as primary complaint;
Chronic tinnitus (for at least 6 months based on history);
Age 18–80 years;
Ability to understand and consent to the research requirements, and to participate (hearing ability, intellectual capacity, no plans for sabbaticals or long-term holidays, no plans for pregnancy);
A score of >22 on the Montreal Cognitive Assessment (MoCA), i.e., adults without mild cognitive impairment ;
Ability and willingness to use the UNITI mobile applications on their smartphones;
A score of ≥18 in the Tinnitus Handicap Inventory (THI);
Willing to use a hearing aid (if there was indication);
If a drug therapy with psychoactive substances (e.g., antidepressants, anticonvulsants) existed at the beginning of the therapeutic intervention, it must have been stable for at least 30 days. The therapy should remain constant during the whole study, but a necessary change was not an exclusion criterion. Any change in medication was documented in the case report form (CRF).
**Exclusion Criteria**
Objective tinnitus/heartbeat-synchronous tinnitus as primary complaint;
Start of any other tinnitus-related treatments, especially hearing aids (HA), structured counselling, sound therapy (with special devices, expecting long term effects) or cognitive behavioural therapy in the last 3 months before the start of the study *;
Otosclerosis/acoustic neuroma or other relevant ear disorders with fluctuating hearing;
Present acute infections (e.g., acute otitis media, otitis externa, acute sinusitis);
Meniere's disease or similar syndromes (but not vestibular migraine);
Serious internal, neurological, or psychiatric conditions;
Epilepsy or other CNS disorders (e.g., brain tumour, encephalitis);
Clinically relevant drug, medication or alcohol abuse up to 12 weeks before the start of the study;
Missing written informed consent;
Severe hearing loss—inability to communicate properly in the course of the study; 70 dB hearing level at 4 kHz (deviations were possible if there was a clinical justification for it);
One deaf ear.

* If an HA has already been worn for three months before screening, eligible candidates were allowed to participate but were automatically assigned to the no HA indication group.

Table S2. Stimulus and acquisition parameters for ABR and AMLR recordings.

ABR			
**Stimulus Parameters**		**Acquisition Parameters**	
Type of transducer	Insert phone	Analysis time	15 ms
Sample rate	30 kHz	Sweeps	4000
Type of stimulus	Click	Mode	Monaural
Polarity	Alternate	Electrode montage	Vertical (Fpz, Cz, M1/M2)
Repetition rate	Stimuli per second: 22 Hz	Filter setting for input amplifier	Low Pass: 1500 Hz; high Pass: 33 Hz, 6 dB per octave
Intensity	80 dB nHL	Preliminary display settings	Low pass: 1500 Hz;high Pass: 150 Hz

Masking	Off		
**AMLR**			
**Stimulus Parameters**		**Acquisition Parameters**	
Type of transducer	Insert phone	Analysis time	150 ms
Sample rate	3 kHz	Sweeps	500
Type of stimulus	2 kHz Tone Burst, Manual window	Mode	Monaural
Duration of stimulus	total of 28 sine waves; rise/fall: 4; plateau: 20	Electrode montage	Vertical (Fpz, Cz, M1/M2)
Polarity	Rarefaction	Filter setting for input amplifier	Low Pass: 1500 Hz; high Pass: 10 Hz, 12 dB per octave
Repetition rate	Stimuli per second: 6.1 Hz	Preliminary display settings	Low pass: 100 Hz; high Pass: 15 Hz
Intensity	70 dB nHL		
Masking	Off		

Table S3. Subjects' age in the two treatment response groups of the ABR waveforms.

	Treatment Response Group	Frequency	Mean	Median	Std. Deviation
age	no improvement	114	54.88	56	11.79
	significant improvement	108	52	55	12.8

Table S4. Subjects' age in the two treatment response groups of the AMLR waveforms.

	Treatment Response Group	Frequency	Mean	Median	Std. Deviation
age	no improvement	103	54.77	56	11.55
	significant improvement	96	52.53	55.5	12.89

Table S5. Subjects' age in the four tinnitus localisation groups of the ABR waveforms.

	Tinnitus Localisation	Frequency	Mean	Median	Std. Deviation
age	bilateral	304	52.35	54	12.43
	head	86	49.28	51	12.24
	non-tinnitus	47	50.74	54	12.15
	unilateral	47	50.74	54	12.15

Table S6. Subjects' age in the four tinnitus localisation groups of the AMLR waveforms.

	Tinnitus Localisation	Frequency	Mean	Median	Std. Deviation
age	bilateral	269	52.62	54	12.44
	head	74	50.08	51.5	12.37

Tinnitus Localisation	Frequency	Mean	Median	Std. Deviation
unilateral	46	50.71	54	12.12
non-tinnitus	45	50.68	54.5	12.27

Table S7. Statistical hearing status analysis in two ABR waveform treatment response groups.

Test used	ABR
Mann-Whitney U-Test	*U=5854, p=.529, r= 0.04*
	The effect size r is: 0.04

Table S8. Statistical hearing status analysis in two AMLR waveform treatment response groups.

Test used	ABR
Mann-Whitney U-Test	*=4640.5, p=.456, r= 0.05*
	The effect size r is: 0.05

Table S9. Statistical hearing status analysis in four ABR waveform tinnitus localisation groups.

Test used	ABR
Kruskal-Wallis Test	*p < 0.01*

Table S10. Statistical hearing status analysis in four ABR waveform tinnitus localisation groups (Dunn-Bonferroni tests).

	p	Adj. p
head - bilateral	.059	.354
**head - unilateral**	**.001**	**.004**
head - non-tinnitus	.339	1
bilateral - unilateral	.014	.083
bilateral - non-tinnitus	.01	.06
**unilateral - non-tinnitus**	**<.001**	**.001**

Adj. p: Values adjusted with Bonferroni correction.

Table S11. Statistical hearing status analysis in four AMLR waveform tinnitus localisation groups.

Test used	AMLR
Kruskal-Wallis Test	*p < 0.01*

Table S12. Statistical hearing status analysis in four AMLR waveform tinnitus localisation groups (Dunn-Bonferroni tests).

	p	Adj. p
head - bilateral	.066	.398
**head - unilateral**	**.001**	**.003**
head - non-tinnitus	.376	1
bilateral - unilateral	.01	.062
bilateral - non-tinnitus	.011	.068
**unilateral - non-tinnitus**	**<.001**	**.001**

Adj. p: Values adjusted with Bonferroni correction.

Table S13. Descriptive statistics regarding the effect of tinnitus laterality on the latency and amplitude components of the ABR waveforms.

**AEP component**	**laterality**	**Mean**	**SD**	#
I peak latency	Bilateral	1.5	0.35	289
	Head	1.52	0.32	75
	non-tinnitus	1.56	0.36	43
	Unilateral	1.45	0.39	44
III peak latency	bilateral	3.67	0.28	292
	head	3.69	0.29	83
	non-tinnitus	3.65	0.27	44
	unilateral	3.71	0.34	42
V peak latency	bilateral	5.53	0.47	304
	head	5.42	0.44	85
	non-tinnitus	5.46	0.47	47
	unilateral	5.42	0.4	47
I peak amplitude	bilateral	0.09	0.1	289
	head	0.09	0.1	75
	non-tinnitus	0.07	0.11	43
	unilateral	0.06	0.08	44
III peak amplitude	bilateral	0.2	0.14	292
	head	0.22	0.11	83
	non-tinnitus	0.21	0.11	44
	unilateral	0.2	0.11	42
V peak amplitude	bilateral	0.2	0.12	304
	head	0.19	0.11	85
	non-tinnitus	0.22	0.1	47
	unilateral	0.19	0.11	47

*(# = number of valid values; SD = Std. Deviation)

Table S14. Statistical differences regarding the effect of tinnitus laterality on the latency and amplitude components of the ABR waveforms.

Test used	Latency			Amplitude		
	I	III	V	I	III	V
Kruskal-Wallis Test	*p=.43*	*p=.758*	*p=.164*	*p=.076*	*p=.21*	*p=.384*

Table S15. Descriptive statistics regarding the effect of treatment response on the latency and amplitude components of the AMLR waveforms.

AEP component	laterality	Mean	SD	#
Na trough latency	bilateral	18.69	3.93	266
	head	19.05	4.05	73
	unilateral	19.56	4.5	46
	non-tinnitus	18.44	4.34	45
Pa peak latency	bilateral	28.14	4.05	269
	head	28.67	5.24	74
	unilateral	28.31	4.35	46
	non-tinnitus	28.39	3.68	45
Nb trough latency	bilateral	39.83	6.17	269
	head	40.48	6.52	74
	unilateral	39.37	6.34	46
	non-tinnitus	40.95	5.04	45
Pb peak latency	bilateral	49.05	7.05	269
	head	49.18	7.23	74
	unilateral	49.05	7.67	46
	non-tinnitus	50.51	6.46	45
Na trough amplitude	bilateral	-0.36	0.28	266
	head	-0.43	0.44	73
	unilateral	-0.42	0.42	46
	non-tinnitus	-0.45	0.33	45
Pa peak amplitude	bilateral	0.42	0.26	269
	head	0.45	0.33	74
	unilateral	0.34	0.31	46
	non-tinnitus	0.44	0.25	45
Nb trough amplitude	bilateral	-0.34	0.24	269
	head	-0.36	0.27	74
	unilateral	-0.33	0.26	46
	non-tinnitus	-0.4	0.27	45
Pb peak amplitude	bilateral	0.24	0.27	269
	head	0.21	0.29	74
	unilateral	0.3	0.35	46
	non-tinnitus	0.23	0.28	45

*(# = number of valid values; SD = Std. Deviation)

Table S16. Statistical differences regarding the effect of treatment response on the latency and amplitude components of the AMLR waveforms.

Test used	Latency				Amplitude			
	Na	Pa	Nb	Pb	Na	Pa	Nb	Pb
Kruskal-Wallis Test	*p=.499*	*p=.981*	*p=.535*	*p=.605*	*p=.314*	*p=.294*	*p=.346*	*p=.411*

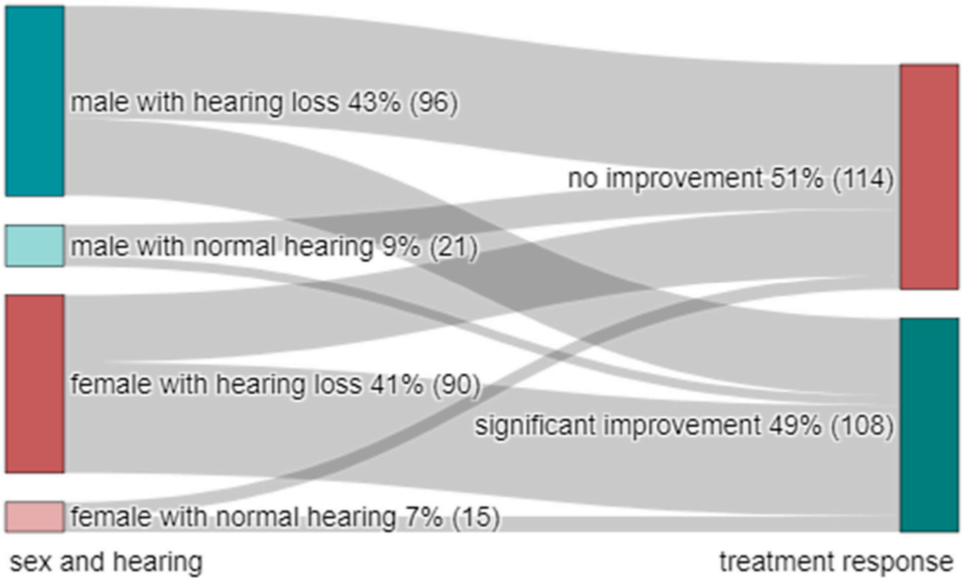

Figure S1. Gender and hearing loss distribution in ABR waveform groups - treatment response.

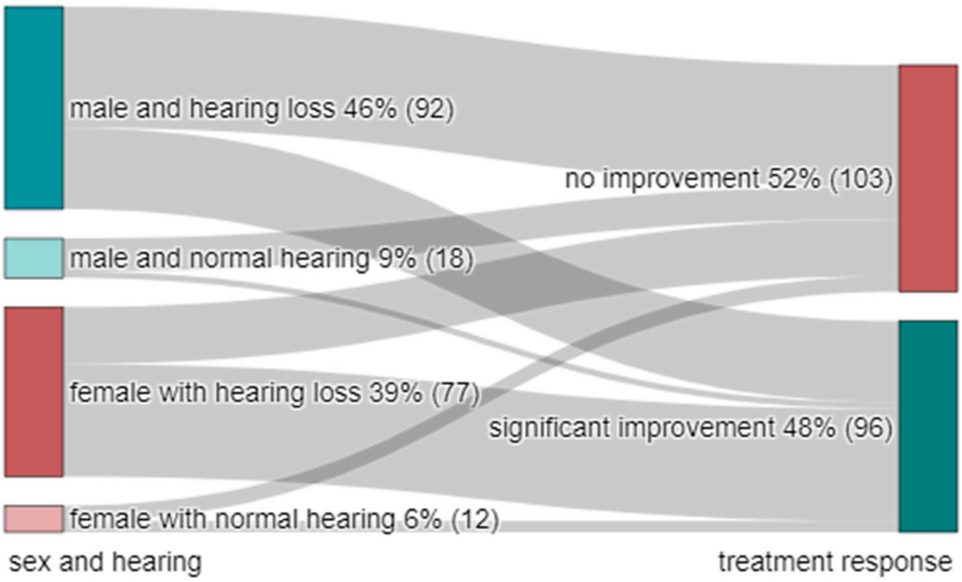

Figure S2. Gender and hearing loss distribution in AMLR waveform groups - treatment response.

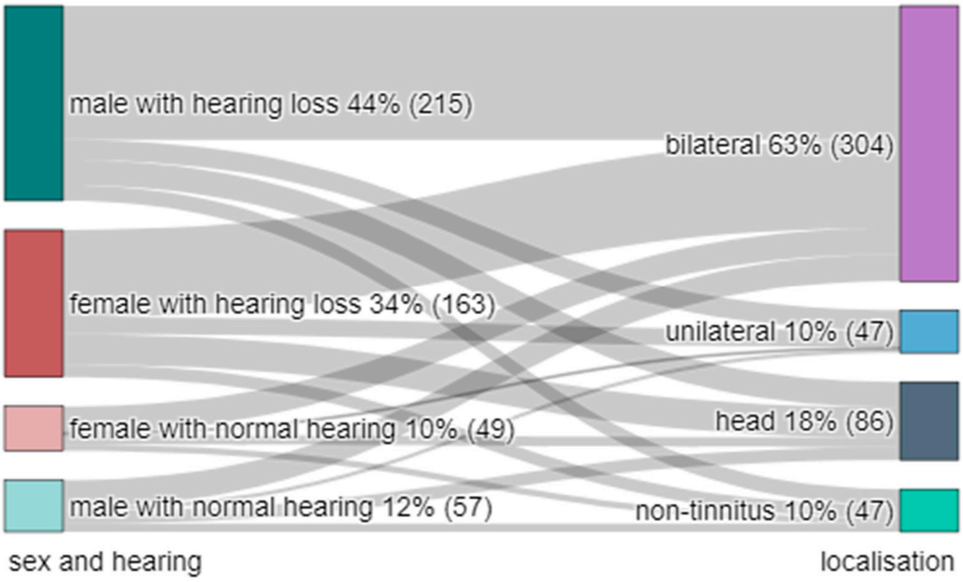

Figure S3. Gender and hearing loss distribution in ABR waveform groups - tinnitus localisation.

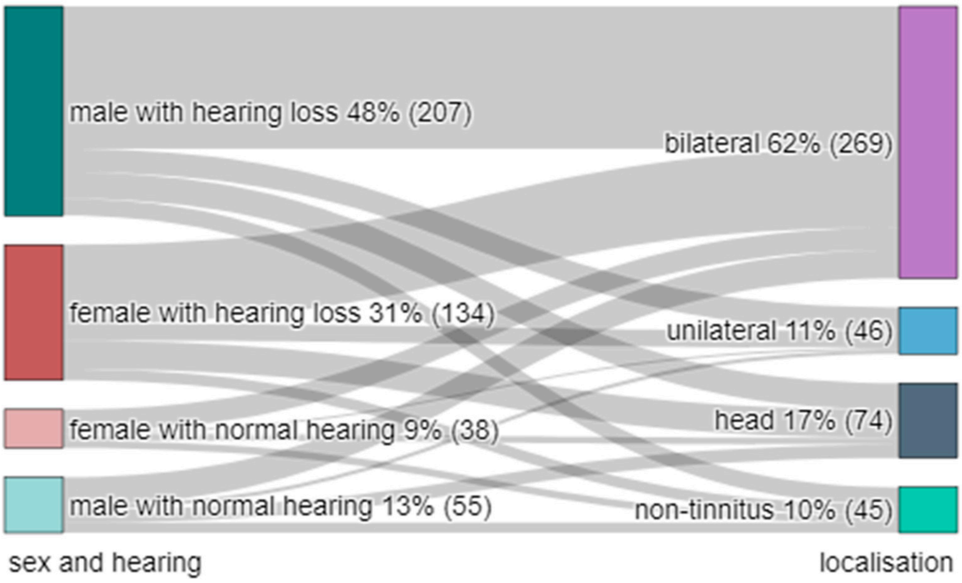

Figure S4. Gender and hearing loss distribution in AMLR waveform groups - tinnitus localisation.
